# Enhanced Photoluminescence and Reduced Dimensionality via Vacancy Ordering in a 10H Halide Perovskite

**DOI:** 10.1021/acs.inorgchem.2c04433

**Published:** 2023-02-13

**Authors:** Hang Liu, Hassan Hafeez, David B. Cordes, Alexandra M. Z. Slawin, Gavin Peters, Stephen L. Lee, Ifor D. W. Samuel, Finlay D. Morrison

**Affiliations:** † EaStCHEM School of Chemistry, University of St. Andrews, North Haugh, St. Andrews KY16 9ST, U.K.; ‡ Organic Semiconductor Centre, School of Physics and Astronomy, SUPA, University of St. Andrews, North Haugh, St. Andrews KY16 9SS, U.K.; § School of Physics and Astronomy, SUPA, University of St. Andrews, North Haugh, St. Andrews KY16 9SS, U.K.

## Abstract

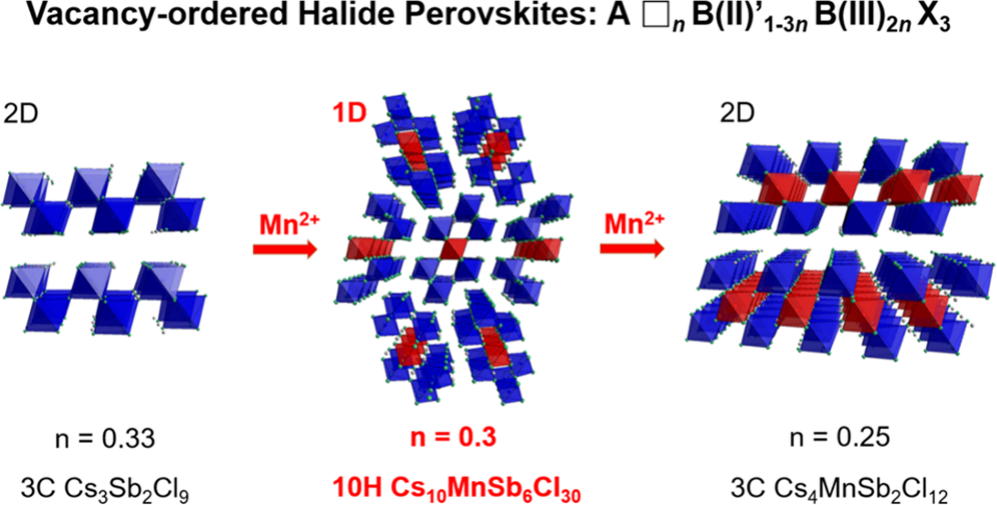

Vacancy-ordered halide perovskites have received great interest in optoelectronic applications. In this work, we report the novel inorganic halide Cs_10_MnSb_6_Cl_30_ with a distinctive 10H (10-layer hexagonal) perovskite polytype structure with (hcccc)_2_ stacking. Cs_10_MnSb_6_Cl_30_ has 30% B-site vacancies ordered at both corner- and face-sharing sites, resulting in [MnSb_6_Cl_30_]^10–^*_n_* columns, i.e., a reduction of octahedral connectivity to 1D. This results in enhanced photoluminescence in comparison to the previously reported 25% vacancy-ordered 3C polytype Cs_4_MnSb_2_Cl_12_ with 2D connectivity. This demonstrates not only the existence of the 10H perovskite structure in halides but also demonstrates the degree of B-site deficiency and stacking sequence variation as a direction to tune the optical properties of perovskite polytypes via vacancy rearrangements.

## Abstract 2

The compound Cs_10_MnSb_6_Cl_30_ is a novel vacancy-ordered halide perovskite with a 10H polytype structure and (hcccc)_2_ stacking. The B-site vacancies order on both face- and corner-sharing sites reducing the octahedral connectivity to 1D, resulting in enhanced photoluminescence compared to related 2D compositions.

## Introduction

In the past decade, both inorganic and organic–inorganic hybrid halide perovskites have generated immense research interest for application in optoelectronic, photocatalytic, and light-emitting diode devices because of their favorable electronic structure, structural tunability, and low cost of fabrication.^1^ For the stoichiometric perovskite with the formula ABX_3_, the B-site cation occupies one quarter of the interlayer octahedral voids formed by the close-packed AX_3_ layers. Several perovskite polytypes exist depending on the close packing sequence, which can be cubic (ABC, e.g., in the prototypical perovskite mineral CaTiO_3_^2^ or the halide CsPbCl_3_^3^), hexagonal (ABA, e.g., BaNiO_3_^4^ or CsNiCl_3_^5^), or a mixture of both. The latter two cases demonstrate the hexagonal perovskite polytypes.^6^

The various perovskite polytypes can be informatively described using a combination of Jagodzinski and Ramsdell notations.^7^ The Jagodzinski notation describes the repeat stacking sequence of cubic (c) and hexagonal (h) close-packed layers, whereas the Ramsdell notation takes the form *n*M where *n* describes the number of layers in the aristotype unit cell and M = C, H, or R indicating cubic, hexagonal, or rhombohedral parent symmetry, respectively. For example, a conventional perovskite formed by three cubic (ccc, i.e., ABC) packed AX_3_ layers is denoted as 3C (e.g., SrTiO_3_^8^), a perovskite with only hexagonal packing (hh, i.e., AB) is denoted as 2H (e.g., BaNiO_3_ and CsNiCl_3_), the hexagonal polytype with four cubic and two hexagonal AX_3_ layers, (cch)_2_, in a hexagonal primitive cell is denoted as 6H (e.g., RbMgF_3_^9^ or BaFeO_3_^10^), and so on. To specifically define each hexagonal polytype, a combination of Ramsdell and Jagodzinski notations is unambiguous, e.g., 10H (hhccc)_2_,^11^ 10H (hhhcc)_2_,^12^ and 10H (hcccc)_2_.^13^

A key feature of these perovskite polytypes is the connectivity of the BX_6_ units, which can be either corner-sharing octahedra (CSO) formed in cubic close-packed stacking or face-sharing octahedra (FSO) generated by hexagonal close-packed stacking. For stoichiometric perovskites, CSO in 3C materials results in three-dimensional (3D) octahedral connectivity, but the 2H (hh) polytype with only FSO exhibits one-dimensional (1D) chains of octahedra. For other hexagonal polytypes, varying degrees of CSO and FSO exist while retaining a 3D network, and this variation of connectivity influences the band structure due to the different B–X orbital interactions in the FSO.^14^ Increasing degrees of FSO generally leads to an increase of the band gap.^15^ For inorganic perovskites, substitution of higher valence B cations is a common strategy to adjust the octahedral connectivity by the introduction of compensating B-site vacancies to maintain electroneutrality.^16^ The preferred occupancy (ordering) of vacancies at specific sites disrupts the connectivity of the 3D octahedral framework, resulting in a reduction of dimensionality to 2D, 1D, or 0D, depending on the relative amount and position of the vacancies. In comparison with 3D counterparts, lower dimensional perovskites have been proven to be important materials with highly efficient light emitting performance and improved moisture stability.^17^

**Figure 1:**
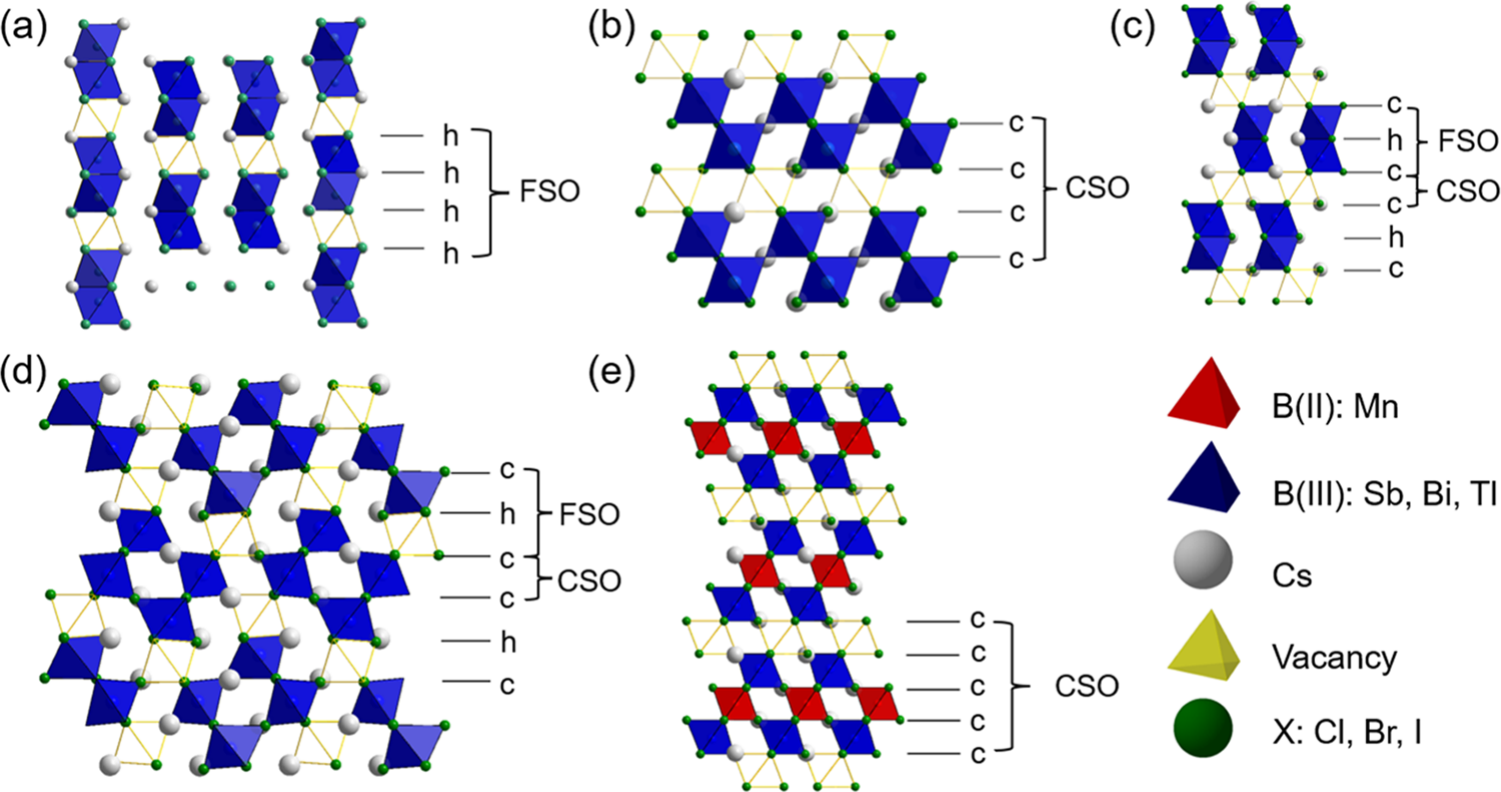
Polyhedral representation of the structures of A_3_B_2_X_9_ B-site-deficient perovskites: (a) 0D 2H (h)_3_ Cs_3_Tl_2_Cl_9_, (b) 2D 3C (c)_3_ Cs_3_Bi_2_Br_9_, (c) 0D 6H (hcc) Cs_3_Bi_2_I_9_, (d) 1D 6H (hcc)_2_ Cs_3_Bi_2_Cl_9_ type, and (e) 2D 3C (c)_4_ Cs_4_MnSb_2_Cl_12_. For ease of comparison, all structures are viewed along the close packing layers.

In the recent years, the B-site-deficient halide perovskites A_3_B(III)_2_X_9_ (A = K, Rb, Cs; B = Sb, Bi, Tl, etc.; and X = Cl^–^, Br^–^, I^–^) have been revisited to investigate the relationship between the crystal and electronic structure (specifically band gap).^18−21^ It is typical for these A_3_B_2_X_9_ compounds that AX_3_ forms close-packed layers with the B cations occupying 2/3 of the octahedral sites with the remaining 1/3 vacant. The most well-defined A_3_B_2_X_9_ structures can be classified into four aristotype structures in relation to the packing sequence of the fully occupied perovskite equivalent and the resulting vacancy ordering as shown in Figure 1. Cs_3_Tl_2_Cl_9_ is derived from the 2H parent with only hexagonal (h)_2_ stacking but with 1/3 B-site vacancies ordered in each FSO layer, resulting in the 0D connectivity of B_2_X_9_ “dimers,” Figure 1a.^22^ In comparison, Cs_3_Bi_2_Br_9_ is based on conventional cubic close-packed 3C (c)_3_ perovskites with vacancies ordered in every third CSO layer to generate a 2D layered structure, Figure 1b.^23^ Both Cs_3_Bi_2_I_9_ (also denoted as Cs_3_Cr_2_Cl_9_ type)^24^ and Cs_3_Bi_2_Cl_9_^25^ have 6H (hcc)_2_ stacking, but the different vacancy ordering results in differing dimensionality of the octahedral connectivity. In Cs_3_Bi_2_I_9_, the vacancies occur only in the corner-sharing octahedral (CSO) sites, resulting in a 0D dimer structure, Figure 1c, which is similar to Cs_3_Tl_2_Cl_9_ but with layering of the dimers in the close packing plane.^26^ In contrast, Cs_3_Bi_2_Cl_9_ has vacancy ordering at the FSO sites with 50% occupancy, instead resulting in 1D connective columns of four octahedra running perpendicular to the closed-packing direction, Figure 1d. These examples neatly demonstrate the opportunity for structural tuning through vacancy ordering.

Recently, a new series of B-site-deficient perovskites with 25% vacancies was reported by Vargas et al. and can be described by the formula A_4_B′(II)B(III)_2_X_12_ (A = Cs^+^, Rb^+^; B′ = Mn^2+^, Cd^2+^, Cu^2+^; B = Sb^3+^, Bi^3+^, In^3+^; and X = Cl^–^, Br^–^).^27−30^ These compounds have an entirely cubic close-packed AX_3_ structure (the same as Cs_3_Bi_2_Br_9_, Figure 1b), but the B-site vacancies order such that every fourth CSO layer is empty rather than every third, Figure 1e. From a general view, both A_3_B_2_X_9_ and A_4_B′B_2_X_12_ belong to special cases of B-site-deficient halide perovskite with the general formula A(□_*n*_B′_1–3*n*_B_2*n*_)X_3_ with *n* = 0.33 and 0.25, respectively (*n* represents the fraction of vacancies per perovskite formula unit). By varying *n*, a series of potential new compounds and structures with varying octahedral connectivity can be generated, which is dependent on the extent and configuration of vacancy distribution as well as the AX_3_ packing sequence of the structure.

In this study, we report the synthesis of a novel 1D halide derivative 10H perovskite Cs_10_MnSb_6_Cl_30_ (*n* = 0.3) with ordered vacancies at both CSO and FSO sites, which displays enhanced photoluminescence compared to the related 2D Cs_4_MnSb_2_Cl_12_ compound. The discovery of this vacancy-ordered 10H halide demonstrates the strategy to tailor the structure and properties of B-cation-deficient perovskites through a combination of vacancy ordering and stacking sequence variation.

## Experimental Methods

### Synthesis

Raw materials, CsCl (99%), MnCl_2_ (97%), SbCl_3_ (99%), MgCl_2_ (99%), and hydrochloric acid in water (37%), were purchased from Alfa Aesar and used without further purification. All other solvents were obtained from commercial routes and used as-received.

Cs_10_MnSb_6_Cl_30_ single crystals (SCs) were prepared by a hydrothermal reaction in a 40 mL stainless steel autoclave. To obtain the required high chloride ion concentration during synthesis while minimizing the amount of HCl used, an excess amount of MgCl_2_ (10 mmol, 0.9521 g) was dissolved in 5 mL of dilute 20% HCl to obtain a transparent solution. Stoichiometric amounts of MnCl_2_ (1 mmol, 0.1258 g) and SbCl_3_ (6 mmol, 1.3688 g) were then dissolved in the solution. To prevent the formation of the secondary phase Cs_3_Sb_2_Cl_9_, a smaller amount of CsCl (1 mmol, 0.1684 g) was finally added to the solution. The pressure vessel was then heated in an oven at 150 °C for 24 h before cooling to room temperature in air. The product was filtered by vacuum filtration, washed with ethanol several times, and dried in a vacuum desiccator overnight.

Cs_10_MnSb_6_Cl_30_ powder was also prepared by solid-state synthesis. Stoichiometric amounts of CsCl (10 mmol, 1.6836 g), MnCl_2_ (1 mmol, 0.1258 g), and SbCl_3_ (6 mmol, 1.3688 g) were mixed and ground together in a Fritsch Pulverisette planetary ball mill at 600 rpm for 2 h using 60 cm^3^ Teflon pots and high-wear-resistant zirconia media. The mixture was then uniaxially pressed into a 10 mm diameter pellet using a stainless steel pellet die under a load of 1 ton and heated at 220 °C in a furnace for 2 h.

Cs_4_MnSb_2_Cl_12_ powder was prepared by a conventional solution route.^27^ Stoichiometric amounts of MnCl_2_ (1 mmol, 0.1258 g) and SbCl_3_ (2 mmol, 0.4562 g) were dissolved in 3 mL of conc. 37% HCl on a hot plate at 90 °C with continuous magnetic stirring. CsCl (1 mmol, 0.1684 g) was then dissolved in 2 mL of conc. 37% HCl with stirring. Cs_4_MnSb_2_Cl_12_ powder was obtained by adding the precursor CsCl solution dropwise to the MnCl_2_ and SbCl_3_ solution. The resulting powder was washed with ethanol and dried under vacuum for 12 h.

### Characterization

Single crystals prepared by hydrothermal reactions and powders synthesized by either a precipitation or solid-state method were characterized by single-crystal and powder X-ray diffraction (SCXRD and PXRD), respectively. X-ray diffraction data for Cs_10_MnSb_6_Cl_30_ were collected using a Rigaku FR-X Ultrahigh Brilliance Microfocus RA generator/confocal optics with an XtaLAB P200 diffractometer [Mo Kα radiation (λ = 0.71073 Å)]. Intensity data were collected using ω steps accumulating area detector images spanning at least a hemisphere of reciprocal space. Details of structural solution are provided in the Supporting Information. PXRD data were obtained using a PANalytical Empyrean diffractometer with Cu Kα1 (λ = 1.5406 Å) and 2θ angle ranging from 3 to 70° at 298 K. Basic Rietveld refinements of PXRD data using GSASII^31^ were performed to confirm phase purity and for the determination of lattice parameters. The structures of all samples were refined using their corresponding structural model obtained from single-crystal data.^27^

Morphology observation and elemental analysis based on energy-dispersive X-ray spectroscopy (EDS) were performed on the single crystals to confirm the composition and stoichiometry and to exclude the presence of magnesium using a JEOL JSM-IT200 scanning electron microscope (SEM) with an accelerating voltage of 15 kV.

Pseudoabsorbances were calculated from steady-state UV–vis diffuse reflectance data recorded using a JASCO-V650 double-beam spectrophotometer based on the Kubelka–Munk transformation.^32^ Tauc plots assuming both direct and indirect transitions were applied to the pseudoabsorbance spectra to estimate the band gap of Cs_10_MnSb_6_Cl_30_. Powders from ground Cs_10_MnSb_6_Cl_30_ SCs and solution-prepared Cs_4_MnSb_2_Cl_12_ were used in the UV–vis diffuse reflectance measurements (see the Supporting Information for more details).

Steady-state emission and excitation spectra of the powder samples were recorded at 298 K using a spectrofluorometer (Edinburgh Instruments FLS980) with a 150 W ozone-free xenon arc lamp as the excitation source. The photoluminescence quantum yield measurement was conducted using an absolute quantum yield spectrometer model C9920-02 (Hamamatsu, Japan) equipped with a xenon light source. A quartz substrate was used to obtain the blank measurement shown in Figures S6 and S7. All PL, PLE, and PLQY measurements were performed on the solid-state synthesized Cs_10_MnSb_6_Cl_30_ and solution-prepared Cs_4_MnSb_2_Cl_12_ powders.

Dielectric measurements and thermogravimetric and differential scanning calorimetry (TG-DSC) analysis were performed to examine the thermal stability and possible phase transition in Cs_10_MnSb_6_Cl_30_ (see the Supporting Information for details).

The magnetic susceptibility of ground SCs Cs_10_MnSb_6_Cl_30_ was measured using a Quantum Design MPMS3 SQUID magnetometer. For magnetic measurements, single crystals were favored over solid-state powders to avoid any risk of signals from impurities picked up from the stainless-steel pellet die during synthesis; note that small Cs_3_Sb_2_Cl_9_ crystals were only present in negligible amounts in the SC samples and in any case is diamagnetic. Approximately 58 mg of samples were enclosed within in a polycarbonate capsule that was then located inside a plastic straw attached to the MPMS3 sample rod. Field-cooled (FC) measurements were applied in the temperature ranging from 2 to 300 K under a DC magnetic field of 100 Oe. Diamagnetic correction was performed on the collected data using Pascal’s constant.^33^

## Results and Discussion

### Chemical and Structural Characterization

**Figure 2:**
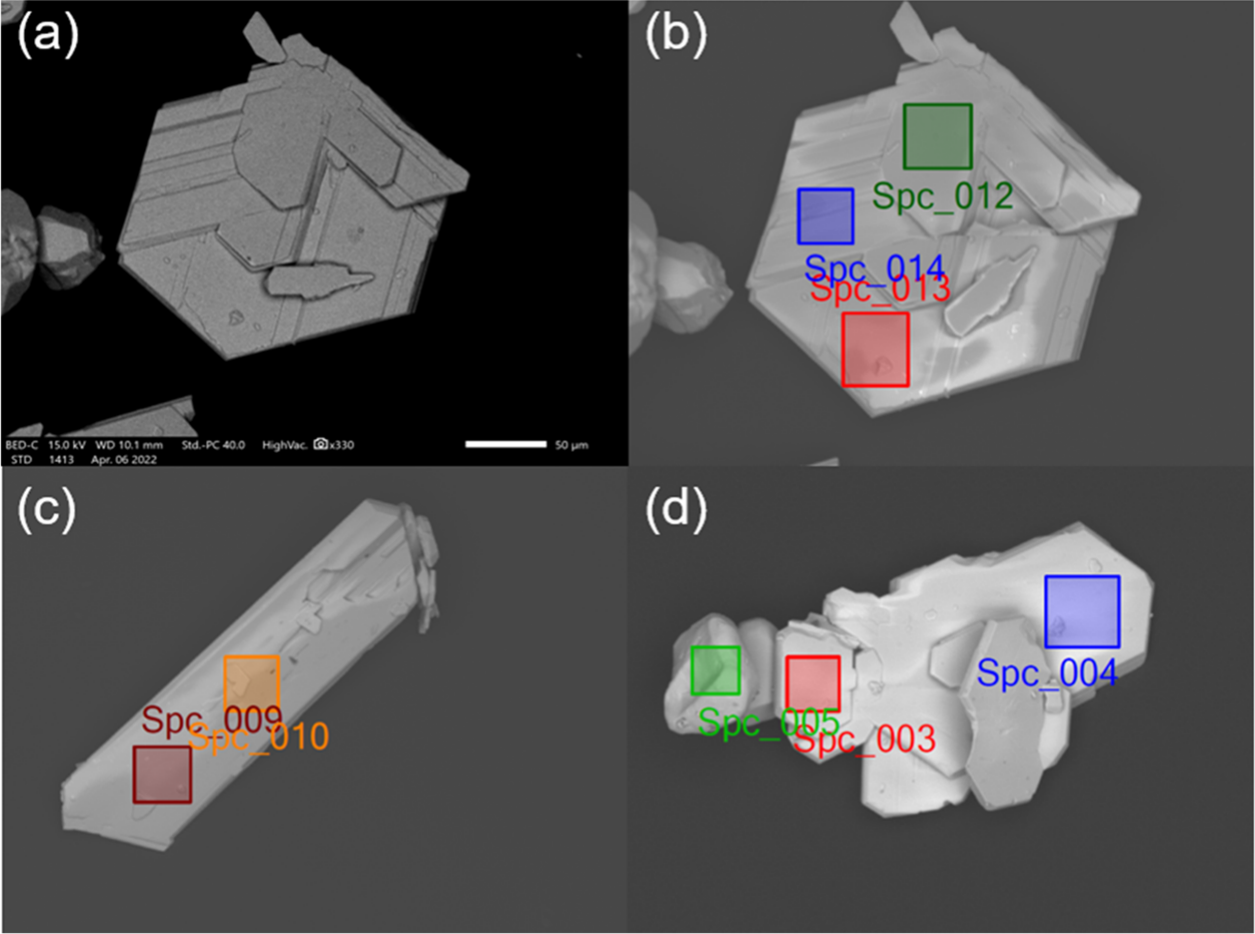
SEM morphologies (secondary electron image, a) and EDS analysis sites (back-scattered electron images, b–d) of Cs_10_MnSb_6_Cl_30_ and secondary phase Cs_3_Sb_2_Cl_9_ single crystals (far left crystal in panel (d), indicated by the Spc_005 data point). The scale bar in panel (a) is 50 μm.

SEM of as-synthesized Cs_10_MnSb_6_Cl_30_ SCs indicated imperfect transparent hexagons with evidence of twin boundaries at the surface as demonstrated in Figure 2. EDS analyses were performed at nine sites on three single crystals of Cs_10_MnSb_6_Cl_30_, confirming the stoichiometry of the crystals. A few smaller single crystals of different morphologies with a clear phase boundary from the main phase Cs_10_MnSb_6_Cl_30_ were observed, Figure 2d; EDS confirms these as Cs_3_Sb_2_Cl_9_, Table 1. It is important to note that these Cs_3_Sb_2_Cl_9_ crystals are present in negligible amounts and were only rarely detected due to the different morphologies; the scarcity of these crystals in samples prepared under hydrothermal conditions means that they were undetectable by all other techniques used in this study. Due to the high Sb^3+^ concentration utilized in the hydrothermal reaction, the cocrystallization of both Cs_3_Sb_2_Cl_9_ and Cs_10_MnSb_6_Cl_30_ is very difficult to inhibit; however, the former was present in only very small amounts as to be undetectable by XRD. Although an excess of MgCl_2_ was added to achieve the required chloride ion concentration, no Mg could be detected in the single crystals by EDS. EDS mapping was also performed at four sites on Cs_10_MnSb_6_Cl_30_ powder prepared by solid-state synthesis as demonstrated in Table 1 and Figure S1.

**Table 1:** Compositional Analysis Using EDS for Cs_10_MnSb_6_Cl_30_ Single Crystals, Secondary (Cs_3_Sb_2_Cl_9_) Phase Crystals, and Cs_10_MnSb_6_Cl_30_ Powder Prepared by Solid-State Synthesis

	Cs (atom %)	Mn (atom %)	Sb (atom %)	Cl (%)
SCs Cs_10_MnSb_6_Cl_30_	21.71 ± 0.12	1.95 ± 0.06	13.90 ± 0.09	62.43 ± 0.19
secondary phase Cs_3_Sb_2_Cl_9_	20.63 ± 0.3	NA	17.18 ± 0.25	62.18 ± 0.46
powder Cs_10_MnSb_6_Cl_30_	22.44 ± 0.12	2.02 ± 0.06	14.04 ± 0.09	61.51 ± 0.19

**Figure 3:**
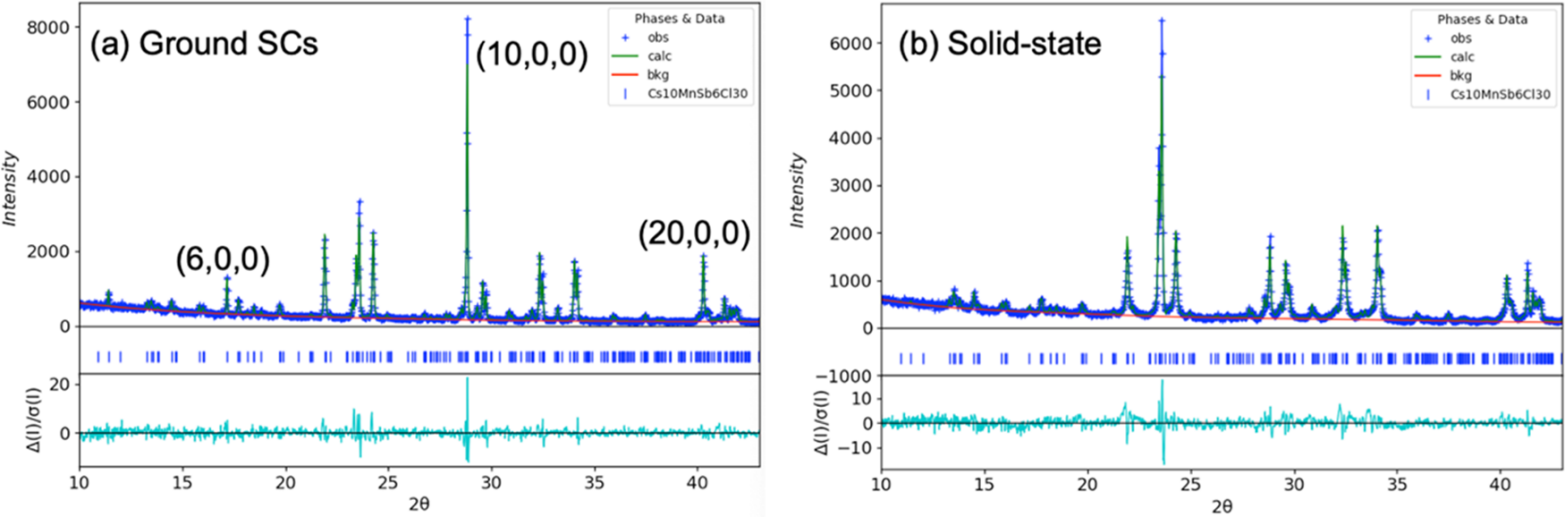
Rietveld refinement profiles for powder X-ray diffraction data of (a) ground single crystals of Cs_10_MnSb_6_Cl_30_ and (b) solid-state synthesized Cs_10_MnSb_6_Cl_30_, confirming the formation of 10H phase as determined from single-crystal XRD.

The crystal structure of Cs_10_MnSb_6_Cl_30_ was determined at 173 K by single-crystal X-ray diffraction (SCXRD), Table 2. Based on the SCXRD data, Cs_10_MnSb_6_Cl_30_ is found as an orthorhombic phase in the space group *Pnnm* (space group #58). Additional data collections were also undertaken at room temperature and 100 K, both of which showed isostructural unit cells. Phase purity and lattice parameters of samples prepared by the various routes were also confirmed by powder X-ray diffraction (PXRD), Figures 3 and S2. Initial PXRD data from crushed single crystals of Cs_10_MnSb_6_Cl_30_ showed systematic intensity variations associated with preferred orientation, and Rietveld refinements were performed after regrinding the single crystals into finer particles. To correlate the preferred orientation in ground single crystals, spherical harmonic functions were employed to refine the texture in the sample as shown in Figure 3a.^34^ The increased intensity of (*h*00)-indexed peaks indicates that the PXRD pattern obtained from ground SCs exhibits crystallographic preferred orientation along the closed-packing (100) direction and is consistent with the plate-like hexagonal-shaped single crystals obtained. There was no evidence of preferred orientation in either the solid-state synthesized Cs_10_MnSb_6_Cl_30_ sample or solution-prepared Cs_4_MnSb_2_Cl_12_. All Cs_10_MnSb_6_Cl_30_ samples appear single phase despite the observation of Cs_3_Sb_2_Cl_9_ single crystals during SEM and EDS analyses; this confirms that this secondary phase is present in only very small amounts as to be undetectable by XRD. The refinements of both solid-state and crushed SC Cs_10_MnSb_6_Cl_30_ samples yield similar lattice parameters.

**Table 2:** Selected Crystallographic Data for Cs_10_MnSb_6_Cl_30_

formula	Cs_10_MnSb_6_Cl_30_
formula weight (g/mol)	3178.04
crystal description	colorless plate
crystal size (mm)	0.09 × 0.06 × 0.01
crystal system	orthorhombic
space group	*Pnnm* (no. 58)
*a* (Å)	30.7287(7)
*b* (Å)	12.99555(3)
*c* (Å)	7.5200(3)
volume (Å^3^)	2993.75(13)
*Z*	2
density (calc., g/cm^3^)	3.526
absorption coefficient (mm^–1^)	10.213
*F*(000)	2782
θ range (deg)	1.706–29.752
no. of reflections collected	52 396
no. of unique reflections (*R*_int_)	8078 (0.0356)
parameters, restraints	129, 0
goodness of fit on *F*^2^	1.015
*R*_1_, *w*R**_2_ [*I* > 2σ(*I*)]	0.0297, 0.0825
*R*_1_, *w*R**_2_ (all data)	0.0409, 0.0863
largest difference peak/hole [e/Å^3^]	2.438, −0.724

From the TGA analysis, it can be observed that Cs_10_MnSb_6_Cl_30_ is thermally stable until 545 K in an inert atmosphere as demonstrated in Figure S5a. From DSC analysis and dielectric spectroscopy data shown in Figures S4 and S5b, there is no evidence of any phase transitions between 50 and 545 K.

**Figure 4:**
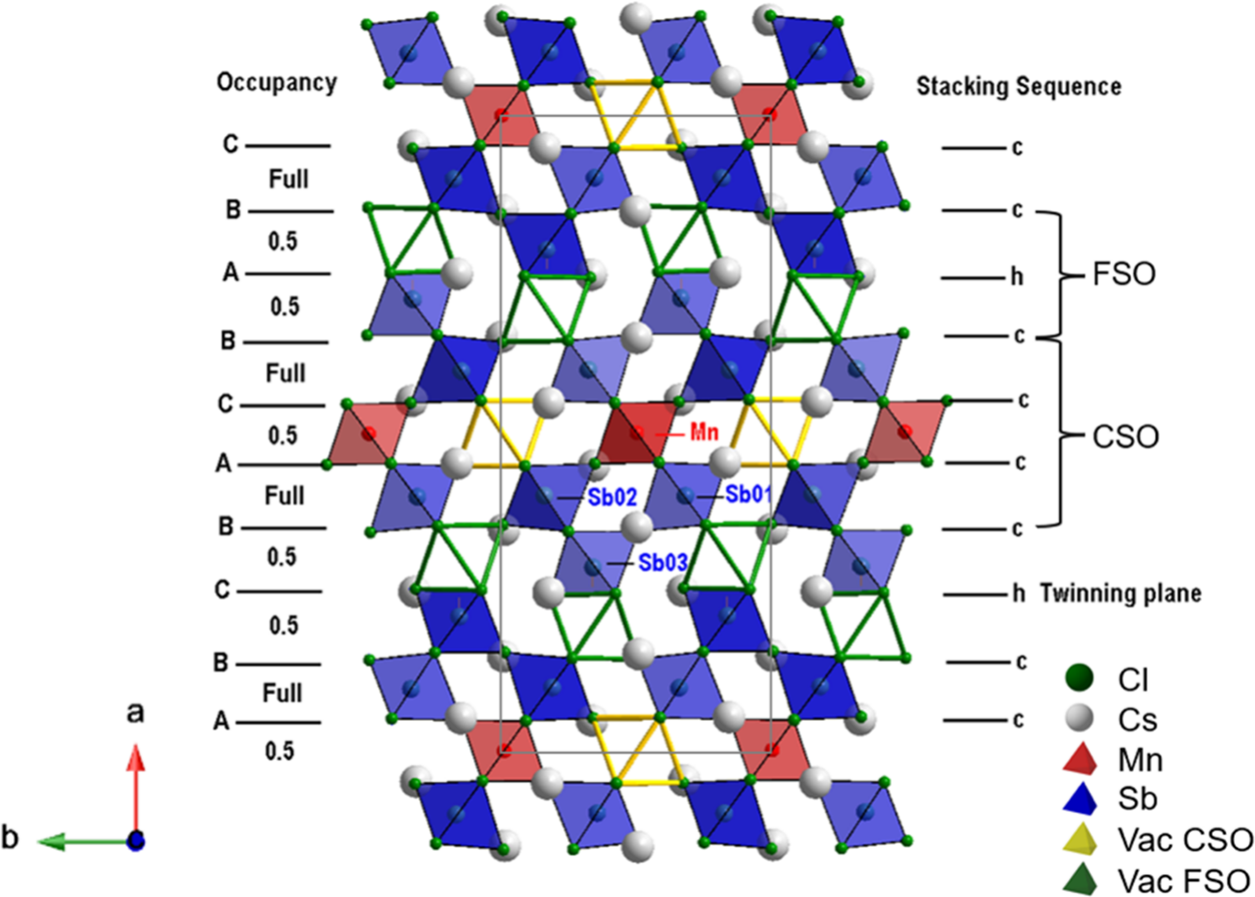
Structure of the vacancy-ordered (hcccc)_2_ type 10H layered Cs_10_MnSb_6_Cl_30_ perovskite viewed along the *c*-axis (in the packing layer plane). To emphasize the underlying 10H parent structure, vacant polyhedra are outlined, with occupied polyhedra shown as shaded.

As illustrated in Figure 4, Cs_10_MnSb_6_Cl_30_ is a 10H perovskite with (hcccc)_2_ stacking but with ordered vacancies in both FSO and CSO octahedral sites. Initially ignoring the vacancies for clarity, the 10H structure can be described by a successive stacking of FSO layers separated by CSO blocks three octahedra deep. The Mn cations selectively occupy only the middle CSO layer within these blocks. Hence, a complete sequence can be viewed as the succession of three CSO blocks and two FSO blocks. An important feature is the vacancy distribution among the five octahedral layers, which leads to a dimensionality reduction from 3D to 1D. Four crystallographic octahedral sites are labeled in Figure 4, where Sb(01) and Sb(02) fully occupy the outer layers of the CSO block and Sb(03) is distributed in an ordered fashion across 50% of the octahedral sites in the FSO block. This distribution results in a distorted “checkerboard” ordering within each layer but with an offset between the layers such that there are no face-sharing Sb_2_Cl_9_ dimers. The Mn^2+^, located in the middle CSO layer, also occupies 50% of the octahedral sites in this layer in a similar checkerboard ordered fashion. From Figure 4, it can be observed that the vacancy ordering in Cs_10_MnSb_6_Cl_30_ can be described by a combination of checkerboard ordering of vacancies in both the half-occupied FSO and Mn-containing CSO layers, and which disrupt the connectivity, resulting in 1D [MnSb_6_Cl_30_]^10–^*_n_* columns running along the *c*-axis of the *Pnnm* structure (*i.e*., orthogonal to the AX_3_ stacking direction).

Vacancy-ordered structures appear to be energetically favorable during aliovalent doping for both close-packed hexagonal halide and oxide perovskites.^16,35,36^ In A_3_B_2_X_9_ halides, the preferred vacant octahedral site is mainly determined by the relative ionic size and electron configuration of B^3+^. For example, in β-Cs_3_Sb_2_Cl_9_,^37^ vacancies tend to occupy the FSO to form a similar “checkerboard” distribution as in Cs_10_MnSb_6_Cl_30_. This is to avoid B–B repulsion in FSO dimers due to the close distance (about 2.943 Å) between two hexagonal packed SbCl_6_ and the poor screening due to the relatively low polarizability of the chloride ion, resulting in the formation of a 1D 6H (hcc)_2_ structure.^38^ However, in Cs_3_Sb_2_I_9_, vacancies prefer to occupy the CSO sequence because of the relatively larger distance (ca. 3.97 Å) and weaker influence of lone pair electrons between two hexagonal packed layers and better screening provided by I^–^, hence yielding a 0D 6H (hcc)_2_ structure. If a cation smaller than Sb is incorporated, such as Cs_3_Cr_2_Cl_9_,^39^ a preferred distribution of vacancies at CSO sites is observed. In comparison with deficient halides, vacancies in analogous B-site-deficient oxides normally preferentially appear in the FSO. For instance, in the similar deficient 10H (hcccc)_2_ Ba_10_Mg_0.25_Ta_7.9_O_30_, the long-range ordering of vacancies at FSO blocks was determined by TEM, although the periodicity of the ordered vacancies is more complex than the zig-zag chains of Cs_10_MnSb_6_Cl_30_ and results in retention of 3D octahedral connectivity.^40^ Compared to 10H oxide analogues with only FSO vacancies, the 10H halide Cs_10_MnSb_6_Cl_30_ contains a higher vacancy ratio of 3/10 with both FSO and CSO vacancies, resulting in a further loss of connectivity to form a 1D structure. Such coexistence of both CSO and FSO vacancies to form isolated 1D columns is rarely observed in either oxide or halide perovskites.

### Magnetic Properties

**Figure 5:**
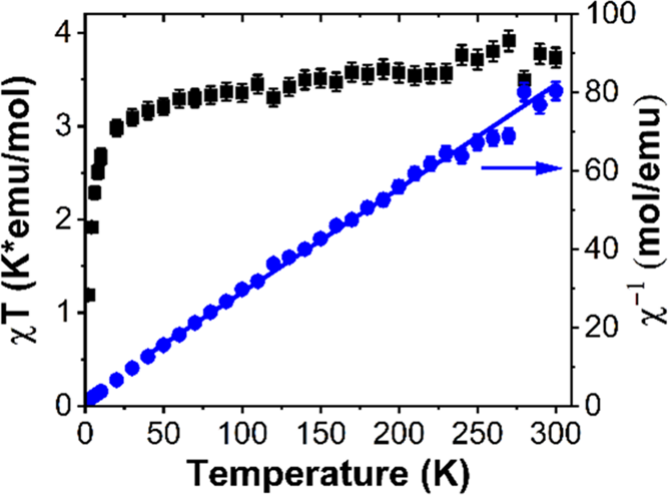
Temperature dependence of magnetic properties for Cs_10_MnSb_6_Cl_30_.

To investigate any magnetic coupling between Mn^2+^ in Cs_10_MnSb_6_Cl_30_, magnetic susceptibility was collected in the temperature range 2–300 K. Despite some noise in the data, presumably due to the low field used and possibly due to some settling of the plate-like single crystals used in the measurement, a linear fitting from 40 to 300 K using the Curie–Weiss law, Figure 5, yields a Weiss constant of −9.43(8) K and an effective magnetic moment of 5.494(5) μ_B_. Although a little low due to data quality as mentioned above, the magnitude of the moment is consistent with the theoretical value 5.916 μ_B_ for spin-only moment of high spin state d^5^ Mn^2+^ and further corroborates the inclusion of Mn^2+^. The negative Weiss constant indicates weak antiferromagnetic coupling between Mn^2+^. Such weak superexchange was also predicted and observed in both 2D Cs_4_MnSb_2_Cl_12_ and Cs_4_MnBi_2_Cl_12_, which is attributed to the four bond Mn–Cl–(Sb/Bi)–Cl–Mn exchange pathway.^27,41^ The product of magnetic susceptibility and temperature is only slowly varying over most of the high temperature range, further supporting the existence of weak antiferromagnetic interactions and a constant effective moment per site.

### Optical Properties

**Figure 6:**
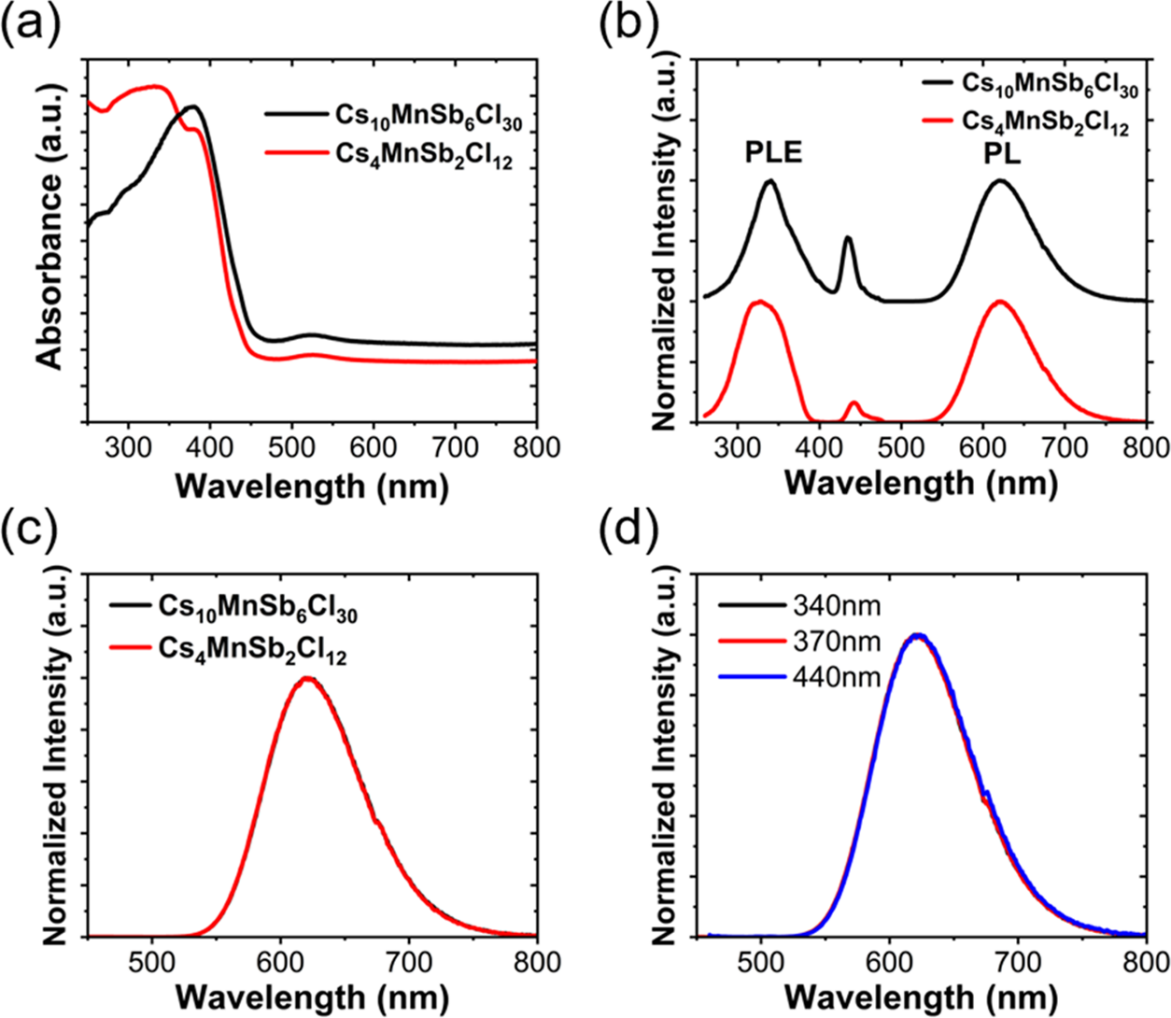
(a) Steady-state absorbance spectra for Cs_4_MnSb_2_Cl_12_ and Cs_10_MnSb_6_Cl_30_; (b) steady-state PL (excited at 340 nm) and PLE (detected at 620 nm) spectra for Cs_4_MnSb_2_Cl_12_ and Cs_10_MnSb_6_Cl_30_ (note the data are offset on the *y*-axis for clarity); (c) PL spectra excited at 340 nm for Cs_4_MnSb_2_Cl_12_ and Cs_10_MnSb_6_Cl_30_; and (d) PL spectra of Cs_10_MnSb_6_Cl_30_ for excitation at 340, 370, and 440 nm.

UV–vis diffuse reflectance of Cs_10_MnSb_6_Cl_30_ was measured and converted into pseudoabsorbance using the Kubelka–Munk (KM) transformation α = (1 – *R*)^2^/2*R*, where α is the absorption coefficient and *R* is the reflectance.^32^ Equivalent data for Cs_4_MnSb_2_Cl_12_ were collected to allow comparison and are shown in Figure 6a. The spectra demonstrate that Cs_10_MnSb_6_Cl_30_ shows similar behavior to Cs_4_MnSb_2_Cl_12_ with the main absorption onset at around 450 nm. Both compounds exhibit weak absorption at about 520 nm, which is assigned to the forbidden ^6^A_1_(S) → ^4^T_1_(G) d–d transition of high spin Mn^2+^.^41,42^ For Cs_4_MnSb_2_Cl_12_, two additional strong absorption peaks are observed at ca. 350 and 380 nm and are ascribed to the partially allowed ^1^S_0_ → ^3^P_1_ transition of Sb^3+^ split by a second-order Jahn–Teller distortion.^43−45^ In comparison with the 3C Cs_4_MnSb_2_Cl_12_ polytype, only one strong, well-defined absorption peak is observed at 380 nm for Cs_10_MnSb_6_Cl_30_, which is also likely due to the ^1^S_0_ → ^3^P_1_ transition of Sb^3+^. Tauc plots of Cs_10_MnSb_6_Cl_30_ were obtained assuming both a direct and indirect band gap, giving values of 2.98 and 2.69 eV, respectively (see the Supporting Information for further details). While indirect band gaps are typically expected for perovskites with adjacent occupied FSO, the combination of the checkerboard ordering in-plane and the zig-zag arrangement between FSO results in no Sb–Sb FSO in this case, and so, the electronic structure is likely to be dominated by CSO orbital interactions. To verify this, DFT calculations need to be performed; these are underway but are complex and time-consuming due to the heavy ions involved and also large supercell volumes associated with the various permutations of the antiferromagnetically ordered ground state. However, considering the structural and compositional similarity to Cs_4_MnSb_2_Cl_12_, which also has no 90° Sb–Cl–Sb interactions, a 2.98 eV direct band gap may be tentatively assumed for Cs_10_MnSb_6_Cl_30_.

Steady-state photoluminescence emission (PL), photoluminescence excitation (PLE), and photoluminescence quantum yield (PLQY) data were collected from both Cs_4_MnSb_2_Cl_12_ and Cs_10_MnSb_6_Cl_30_ powders at room temperature. The PL spectra show wide peaks centered at 620 nm with a full width at half-maximum (FWHM) of ca. 88 nm as illustrated in Figure 6b,c, indicating that the same PL mechanism attributed to the octahedrally coordinated Mn^2+^ transition from ^4^T_1_ to ^6^A_1_ for both Cs_10_MnSb_6_Cl_30_ and the previously reported Cs_4_MnSb_2_Cl_12_.^27^ In addition to a peak at 340 nm (which we attribute to the onset of ^1^S_0_ → ^3^P_1_ transition of Sb^3+^), the PLE data of Cs_10_MnSb_6_Cl_30_ show a strong peak at 440 nm, which corresponds to the ^6^A_1_(S) → ^4^T_2_(G) transitions of high spin Mn^2+^.^45^ However, the emission is independent of excitation wavelength (Figure 6d), indicating that the same species is likely to be responsible for the emission under excitation wavelengths from 340 to 440 nm. In Cs_4_MnSb_2_Cl_12_, the 440 nm feature in PLE is smaller and the emission spectrum is the same for all excitation wavelengths (Figures 6b and S8). The Cs_10_MnSb_6_Cl_30_ samples exhibit larger PLQYs than Cs_4_MnSb_2_Cl_12_. The PLQY of Cs_10_MnSb_6_Cl_30_ was 3.5±1% for excitation at 340 nm and 12.5 ± 1% for excitation at 440 nm (Table 3). For Cs_4_MnSb_2_Cl_12_, the PLQY was 1.3 ± 1% for both excitation wavelengths. The higher PLE and PLQY of Cs_10_MnSb_6_Cl_30_ may suggest that the forbidden d–d transition (^4^T_1_ to ^6^A_1_) may be increasingly allowed due to a symmetry reduction of the MnCl_6_ octahedra as a result of a local distortion; however, further experiments are required to clarify this and, in particular, the reason for the higher PLQY under 440 nm excitation.

**Table 3:** Average Quantum Yield Excited at 340 and 440 nm

	average PLQY (%)	
excitation wavelength (nm)	340 nm	440 nm
Cs_4_MnSb_2_Cl_12_	1.3 ± 1	1.3 ± 1
Cs_10_MnSb_6_Cl_30_	3.5 ± 1	12.5 ± 1

## Conclusions

In conclusion, a novel one-dimensional perovskite Cs_10_MnSb_6_Cl_30_ with a unique vacancy-ordered 10H structure has been prepared and is the first reported 10H halide perovskite. In comparison to the vacancy-ordered 3C Cs_4_MnSb_2_Cl_12_ phase, which displays 2D (layered) octahedral connectivity, the specific vacancy ordering in the 10H Cs_10_MnSb_6_Cl_30_ compound further reduces this dimensionality to 1D (columnar). As a result, the 10H-type Cs_10_MnSb_6_Cl_30_ exhibits stronger photoluminescence and improved PLQY compared to the reported 3C-type Cs_4_MnSb_2_Cl_12_. The current study highlights structural tuning via vacancy ordering as a mechanism to obtain low-dimensional halide perovskites with enhanced optical properties.

## Data Availability

The research data underpinning this publication can be accessed at https://doi.org/10.17630/51767b58-94a9-4ab2-9f93-c4c7edc4fdd0 [ref (46)].

## Supporting Information Available

Materials characterization methods and data including compositional analysis; single-crystal and powder X-ray diffraction; UV–VIS, TGA–DCS, and dielectric data; photoluminescence and quantum yield data (PDF)
