# Supplementary material for: Enhanced Photoluminescence and Reduced Dimensionality via Vacancy Ordering in a 10H Halide Perovskite
Source: Inorg Chem. 2023 Feb 13;62(8):3629–36. doi: 10.1021/acs.inorgchem.2c04433 (PMC9976281; doi:10.1021/acs.inorgchem.2c04433)
Supplement: Supplementary file 1 — ic2c04433_si_001.pdf [file ic2c04433_si_001.pdf]

Supporting information for:

# Enhanced photoluminescence and reduced dimensionality via vacancy ordering in a 10H halide perovskite.

Hang Liu[1], Hassan Hafeez[2], David B. Cordes[1], Alexandra M. Z. Slawin,[1] Gavin Peters,[1] Stephen Lee[3], Ifor D.W. Samuel[2] and Finlay D. Morrison[1,*]

[1] EaStCHEM School of Chemistry, University of St Andrews, North Haugh, St Andrews KY16 9ST, UK.

[2] Organic Semiconductor Centre, SUPA, School of Physics and Astronomy, University of St Andrews, North Haugh, St Andrews KY16 9SS, UK.

[3] School of Physics and Astronomy, SUPA, University of St. Andrews, North Haugh, St. Andrews KY16 9SS, UK.

*email: finlay.morrison@st-andrews.ac.uk

## Table of Contents

Material Characterisation ........ 2

Energy Dispersive X-ray Spectroscopy (EDS) Analysis ........ 2

Single Crystal X-ray Diffraction Studies (SCXRD) ........ 2

Powder X-ray Diffraction Studies (PXRD) ........ 3

Ultraviolet–Visible Reflectance Spectroscopy ........ 4

Dielectric Measurements ........ 4

Thermogravimetric and Differential Thermal Analysis (TGA-DSC) ........ 5

Photoluminescence quantum yield (PLQY) Measurements ........ 6

Photoluminescence Emission (PL) and Excitation (PLE) Measurements ........ 7

References ........ 7

# Material Characterisation

## Energy Dispersive X-ray Spectroscopy (EDS) Analysis

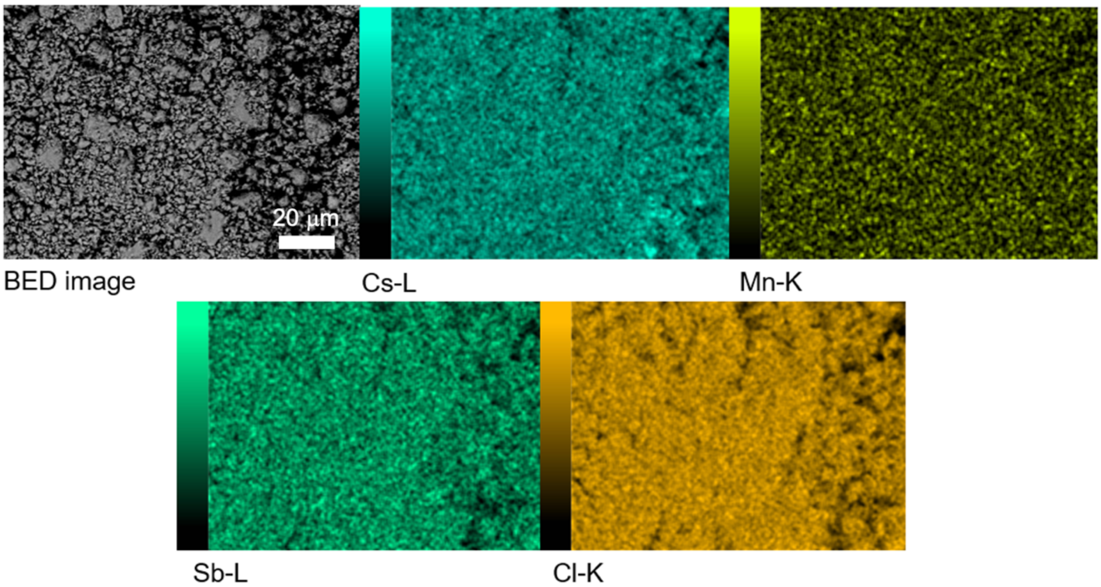


Fig. S1 Back scattered electron (BED) image and EDS elemental maps for solid state synthesised $Cs_{10}MnSb_6Cl_{30}$ powder. The magnification is the same for all images and the scale bar in the BED image indicates 20 microns.

## Single Crystal X-ray Diffraction Studies (SCXRD)

X-ray diffraction data for $Cs_{10}MnSb_6Cl_{30}$ were collected using a Rigaku FR-X Ultrahigh Brilliance Microfocus RA generator/confocal optics with XtaLAB P200 SCXmini CCD diffractometer [Mo Kα radiation (λ = 0.71073 Å)]. Intensity data were collected using ω steps accumulating area detector images spanning at least a hemisphere of reciprocal space. Diffraction data were collected on crystals at room temperature, 173K and 100K, and from the indexing, there was no change in structure with temperature. Following processing, the data collected at 173K was identified as the best quality, so is presented here. Data were collected using CrystalClear[1] and processed (including correction for Lorentz, polarization and absorption) using CrysAlisPro.[2] During indexing of the data, it was clear that the data were from a non-merohedrally twinned crystal. Both unit cells were identified, and data-processing took the presence of both cells into account. The twin-law relating the first cell to the second is [-1.0000 -0.0034 0.0041 -0.0006 0.5062 -1.4835 0.0001 -0.5011 -0.5059], and the refined fraction of the second cell is 0.2899(8). The structure was solved by dual-space methods (SHELXT)[3] and refined by full-matrix least-squares against $F^2$ (SHELXL-2018/3),[4] with anisotropic refinement of all atoms. All calculations were performed using the Olex2 interface.[5] Deposition number 2194265 contains the supplementary crystallographic data for this paper. These data are provided free of charge by the joint Cambridge Crystallographic Data Centre and Fachinformationszentrum Karlsruhe Access Structures service www.ccdc.cam.ac.uk/structures.

## Powder X-ray Diffraction Studies (PXRD)

For Rietveld refinements, the background was refined using a Chebyschev function and a modified pseudo-Voigt profile function was used for peak fitting and subsequent determination of lattice parameters. For the PXRD patterns of both $Cs_{10}MnSb_6Cl_{30}$ samples, data near 44.5° 2θ were excluded from refinement due to the presence of weak reflections from the sample holders.

Table S1 Summary of Rietveld analysis of PXRD data obtained from crushed $Cs_{10}MnSb_6Cl_{30}$ single crystals (SCs), $Cs_{10}MnSb_6Cl_{30}$ powder prepared by solid state. Data for $Cs_4MnSb_2Cl_{12}$ powder is included for comparison.

| Sample | Ground SC $Cs_{10}MnSb_6Cl_{30}$ | Solid state $Cs_{10}MnSb_6Cl_{30}$ | $Cs_4MnSb_2Cl_{12}$ |
|---|---|---|---|
| System | Orthorhombic | Orthorhombic | Trigonal |
| Space group | *Pnnm* | *Pnnm* | $R\bar{3}m$ |
| $a$ (Å) | 30.9119(9) | 30.9189(18) | 7.5545(2) |
| $b$ (Å) | 13.0154(4) | 13.0118(7) | |
| $c$ (Å) | 7.5747(3) | 7.5732(4) | 36.6017(5) |
| $V$ (Å$^3$) | 3047.51(9) | 3046.77(8) | 1809.00(1) |
| w$R_p$ | 0.0856 | 0.0850 | 0.0887 |
| $\chi^2$ | 2.63 | 2.83 | 2.68 |
| GOF | 1.62 | 1.68 | 1.64 |

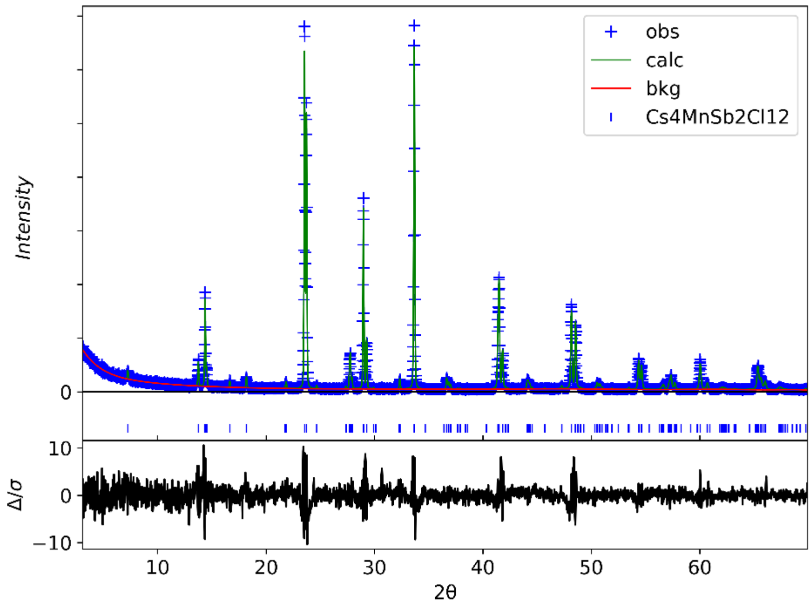


Fig. S2 Rietveld refinement profile for solution prepared $Cs_4MnSb_2Cl_{12}$.

## Ultraviolet–Visible Reflectance Spectroscopy

The reflectance spectra were collected and converted to pseudo absorbance spectra by using the Kubelka-Munk transformation:

$$\alpha = \frac{(1-R)^2}{2R} \tag{1-1}$$

where α is the pseudo absorbance and $R$ the reflectance.[6] To estimate the band gap of $Cs_{10}MnSb_6Cl_{30}$, the Tauc plot is applied based on the relationship between absorbed photon and band gap:

$$(ahv)^{\frac{1}{n}} = A(hv - E_g) \tag{1-2}$$

where $h$ is the Planck's constant, $v$ is the frequency of absorbed photon, $E_g$ denotes band gap and the value of the exponent represents the nature of the electronic excitation.[7] For a direct allowed transition, the value of n is equal to 0.5. For an indirect allowed transition, the value of n is equal to 2. Tauc plots assuming both direct and indirect transitions were applied to the pseudo absorbance spectra of $Cs_{10}MnSb_6Cl_{30}$ as Fig S3 illustrates.

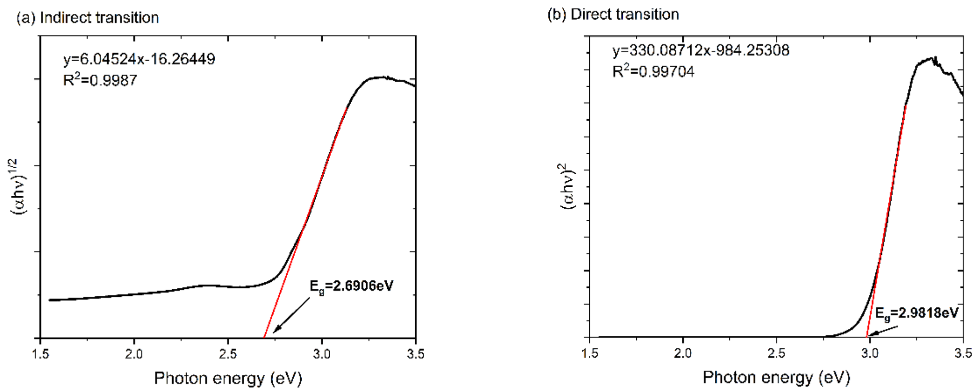


Fig. S3 Tauc plots of the steady-state absorption data for $Cs_{10}MnSb_6Cl_{30}$ (from Figure 6a, main text) assuming (a) indirect transition and (b) direct transition.

## Dielectric Measurements

Dielectric measurements were performed on a pellet of $Cs_{10}MnSb_6Cl_{30}$ *ca.* 1.5 mm thick and 10 mm in diameter, prepared from solid state synthesised powder under uniaxial loading of *ca.* 1 ton. Silver electrodes were coated on the opposing pellet surfaces and dried in a drying oven at 120 °C for 1 h. Dielectric data was collected over the frequency range 100 Hz to 10 MHz with an applied AC electric field of 100mV using an Agilent 4294A in the temperature range 40 K to 475 K. Typical dielectric permittivity curves at 10 kHz and 1 MHz are shown in Fig. S4. The increase in the 10 kHz data at the highest temperatures are due to the encroaching polarisation associated with the sample-electrode interface. The discontinuity in the data at 300 K is due to the change in sample

environment for sub- and above-ambient data. No correction to the data was made to take the change in sample environment into account.

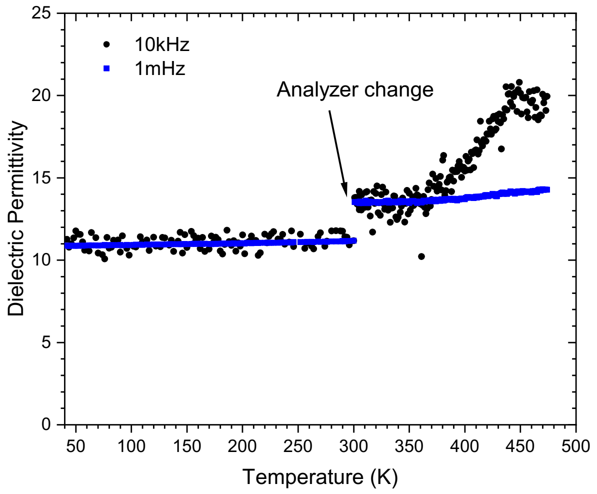


Fig. S4 Dielectric permittivity (at 10 kHz and 1 MHz) as a function of temperature for a $Cs_{10}MnSb_6Cl_{30}$ pellet.

## Thermogravimetric and Differential Thermal Analysis (TGA-DSC)

Thermogravimetric and differential scanning calorimetry analysis (TG-DSC) was performed using a NETZSCH STA 449 F5 system in argon (flow rate 20 ml/min) at a heating rate of 5 K/min. The temperature range was from 308 K to 673 K. TG and DSC analysis for $Cs_{10}MnSb_6Cl_{30}$ solid state synthesised powder is shown in Fig. S5 (a) and (b) respectively. The initial increase at about 308K in both curves is caused by the influx of argon.

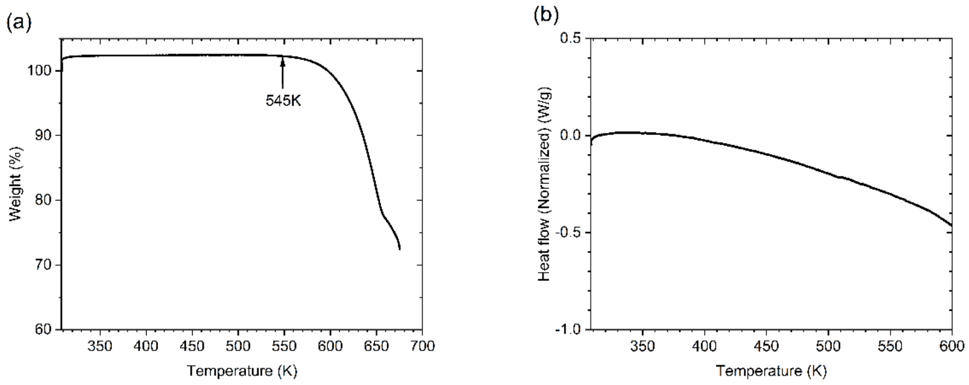


Fig. S5 (a) TG analysis and (b) DSC analysis on solid state synthesized $Cs_{10}MnSb_6Cl_{30}$

## Photoluminescence quantum yield (PLQY) Measurements

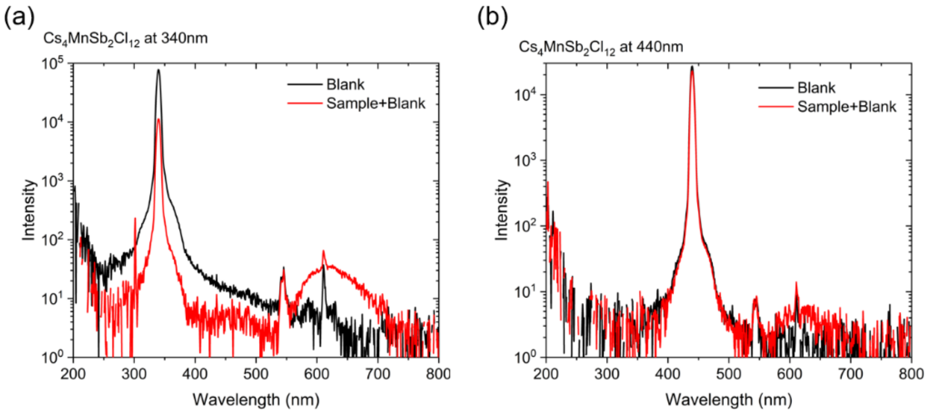


Fig. S6 Quantum yield measurement for $Cs_4MnSb_2Cl_{12}$ excited at (a) 340nm and (b) 440nm. Note the semi-logarithmic y-axis.

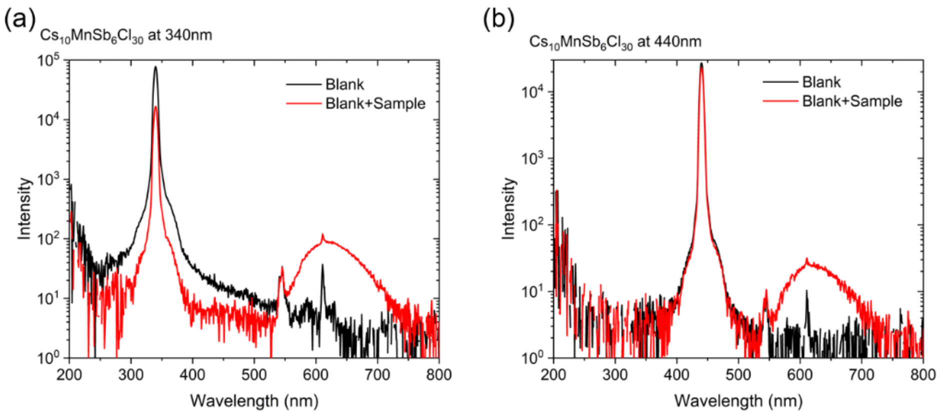


Fig. S7 Quantum yield measurement for $Cs_{10}MnSb_6Cl_{30}$ excited at (a) 340nm and (b) 440nm. Note the semi-logarithmic y-axis.

**Photoluminescence Emission (PL) and Excitation (PLE) Measurements**

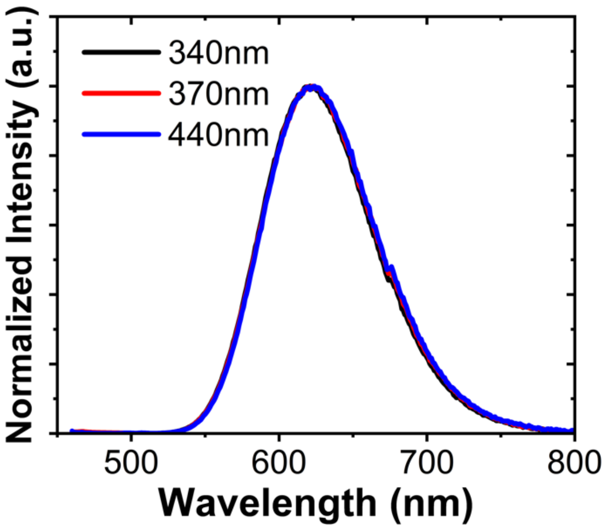


Fig. S8 PL spectra of $Cs_4MnSb_2Cl_{12}$ excited at 340 nm, 370 nm and 440 nm

# References

1. *CrystalClear-SM Expert v2.1*, Rigaku Americas, The Woodlands, Texas, USA and Rigaku Corporation, Tokyo, Japan **2015**.

2. *CrysAlisPro v1.171.41.93a*, Rigaku Oxford Diffraction, Rigaku Corporation, Oxford, U.K. **2020**.

3. Sheldrick, G. M., SHELXT–Integrated space-group and crystal-structure determination. *Acta Crystallogr. A: Found. Adv.* **2015,** *71* (1), 3-8.

4. Sheldrick, G. M., Crystal structure refinement with SHELXL. *Acta Crystallogr. C Struct. Chem.* **2015,** *71* (1), 3-8.

5. Dolomanov, O. V.; Bourhis, L. J.; Gildea, R. J.; Howard, J. A.; Puschmann, H., OLEX2: a complete structure solution, refinement and analysis program. *J. Appl. Crystallogr.* **2009,** *42* (2), 339-341.

6. Makula, P.; Pacia, M.; Macyk, W., How To Correctly Determine the Band Gap Energy of Modified Semiconductor Photocatalysts Based on UV-Vis Spectra. *J. Phys. Chem. Lett.* **2018,** *9* (23), 6814-6817.

7. Viezbicke, B. D.; Patel, S.; Davis, B. E.; Birnie, D. P., Evaluation of the Tauc method for optical absorption edge determination: ZnO thin films as a model system. *Phys. Status Solidi B* **2015,** *252* (8), 1700-1710.
